# Mechanisms of inflammatory microenvironment formation in cardiometabolic diseases: molecular and cellular perspectives

**DOI:** 10.3389/fcvm.2024.1529903

**Published:** 2025-01-14

**Authors:** Menghua Liu, Rumeng Chen, Zhiwei Zheng, Shuling Xu, Chunyan Hou, Yining Ding, Mengling Zhang, Meihua Bao, Binsheng He, Sen Li

**Affiliations:** ^1^ School of Life Sciences, Beijing University of Chinese Medicine, Beijing, China; ^2^ School of Stomatology, Changsha Medical University, Changsha, China; ^3^ Hunan key Laboratory of the Research and Development of Novel Pharmaceutical Preparations, School of Pharmaceutical Science, Changsha Medical University, Changsha, China

**Keywords:** cardiometabolic diseases, inflammatory microenvironment, atherosclerosis, hypertension, diabetic cardiomyopathy

## Abstract

Cardiometabolic diseases (CMD) are leading causes of death and disability worldwide, with complex pathophysiological mechanisms in which inflammation plays a crucial role. This review aims to elucidate the molecular and cellular mechanisms within the inflammatory microenvironment of atherosclerosis, hypertension and diabetic cardiomyopathy. In atherosclerosis, oxidized low-density lipoprotein (ox-LDL) and pro-inflammatory cytokines such as Interleukin-6 (IL-6) and Tumor Necrosis Factor-alpha (TNF-α) activate immune cells contributing to foam cell formation and arterial wall thickening. Hypertension involves the activation of the renin-angiotensin system (RAS) alongside oxidative stress-induced endothelial dysfunction and local inflammation mediated by T cells. In diabetic cardiomyopathy, a high-glucose environment leads to the accumulation of advanced glycation end products (AGEs), activating the Receptor for Advanced Glycation Endproducts (RAGE) and triggering inflammatory responses that further damage cardiac and microvascular function. In summary, the inflammatory mechanisms in different types of metabolic cardiovascular diseases are complex and diverse; understanding these mechanisms deeply will aid in developing more effective individualized treatment strategies.

## Introduction

Cardiometabolic diseases (CMD) represent a complex cluster of disorders, primarily encompassing atherosclerosis, coronary artery disease, and hypertension, all of which are intricately linked to metabolic dysfunction (1–3). These diseases are frequently associated with metabolic syndrome (MetS), a condition characterized by a constellation of symptoms including central obesity, insulin resistance, hyperglycemia, dyslipidemia, and hypertension (4–7). As societies continue to urbanize and modernize, the adverse lifestyle practices have become increasingly prevalent, leading to a rising incidence of cardiometabolic diseases globally (8–11). According to a large number of relevant literature findings, CMD have become one of the leading causes of mortality worldwide, exerting a considerable burden on individuals, families, and public health systems (12, 13). Given that these diseases often progress insidiously, with many patients remaining asymptomatic until severe cardiovascular events such as myocardial infarction, heart failure, or stroke occur, there is an urgent need to unravel the underlying mechanisms of CMD to develop effective preventive and therapeutic strategies that can improve patient outcomes and quality of life (14, 15).

The inflammatory microenvironment plays a critical role in the onset and progression of CMD (16–18). A growing body of research indicates that chronic low-grade inflammation in the cardiovascular system is a pivotal driver of the onset and progression of these diseases (19, 20). Inflammation impacts cardiovascular health through its regulation of processes such as lipid uptake, glucose metabolism, endothelial function, and vascular remodeling (21–24). During the development of atherosclerosis, inflammatory cells such as macrophages, T cells, and mast cells, along with various inflammatory mediators they secrete—including cytokines and chemokines—contribute to the formation and progression of arterial plaques (25, 26). These cells and molecules form a complex inflammatory network that affects multiple aspects of the cardiovascular system, not only causing direct damage to blood vessel walls but also modulating metabolic functions and activating immune responses (17, 26). Despite substantial evidence supporting the critical role of inflammation in CMD, the precise mechanisms by which it is initiated and sustained, and how it interacts with metabolic processes to accelerate disease progression, remain inadequately understood and warrant further investigation.

To address these challenges, this review aims to systematically explore the molecular mechanisms and cellular foundations underpinning the development and maintenance of the inflammatory microenvironment in cardiometabolic diseases. By synthesizing current research advancements, the article seeks to elucidate the specific roles of various inflammatory mediators in CMD, and how their interplay with metabolic abnormalities influences disease progression and outcomes. As our understanding of these pathological mechanisms deepens, we hope to lay a robust scientific foundation for future therapeutic innovations designed to improve the prognosis of patients with cardiometabolic diseases and alleviate the global health burden they pose. By enhancing our understanding of the pathological mechanisms at play, we aim to pave the way for the development of innovative interventions that improve patient outcomes and reduce the global burden of cardiometabolic diseases.

## Metabolic factors are involved in the occurrence and development of cardiovascular diseases

Cardiovascular disease (CVD) is the leading cause of death worldwide and is characterized by atherosclerosis, endothelial dysfunction, inflammation, and oxidative stress (27–30). Metabolic factors play a crucial role in the onset and progression of CVD, primarily including hyperlipidemia, hyperglycemia, and insulin resistance (31). Mendelian randomization (MR) is an analytical method that employs genetic variants as instrumental variables and can be widely used to study the causal associations between exposures and outcomes (32–35). In a MR study, MetS exhibits significant causal relationships with various CVD (36). In clinical trials, patients with MetS have a significantly higher risk of developing CVD compared to those without MetS (37). In patients with hypopituitarism, the high prevalence of MetS is primarily linked to abdominal fat deposition, dyslipidemia, and insulin resistance, with growth hormone replacement therapy showing benefits for lipid profiles and body fat but potentially transiently worsening glucose tolerance (38).

## Recent molecular and cellular mechanisms linking inflammation and metabolism

The interplay between inflammation and metabolism is crucial for physiological balance and disease pathogenesis, including type 2 diabetes (T2D), cardiovascular diseases, obesity, and autoimmune disorders (39). Silent Information Regulator 2 Ortholog 1 (SIRT1) regulates inflammation through various pathways, including affecting metabolic pathways, inflammatory cells and mediators, as well as key signaling pathways, thus playing a significant role in metabolic and immune diseases and serving as a potential therapeutic target (40). Research indicates that small dense low-density lipoprotein-cholesterol (sdLDL-C) and remnant-like particle cholesterol (RLP-C) induce inflammation by activating immune cells, while hyperlipidemia exacerbates inflammatory responses, leading to dysregulated lipid metabolism and alterations in lipoprotein profiles (41). These insights provide new directions for developing targeted therapies for chronic inflammatory and metabolic disorders, potentially leading to more precise and effective treatments.

By targeting these key biological processes, it is possible to develop more precise and effective interventions to improve patient health in the future. Therefore, an in-depth understanding of the mechanisms underlying the formation of the inflammatory microenvironment is essential for disease prevention and treatment. This review will focus on the formation mechanism of the inflammatory microenvironment of atherosclerosis, hypertension and diabetic cardiomyopathy from the perspective of molecular and cellular levels, focusing on the specific factors affecting the inflammatory response.

## Inflammatory mechanisms in atherosclerosis

### Initial events and the role of oxidized Low-density lipoprotein

Atherosclerosis is a chronic inflammatory disease characterized by the formation of lipid deposits, immune cell infiltration, and fibrosis in the arterial wall, eventually leading to atherosclerotic plaque formation (42, 43). One of the initial events in atherosclerosis is the accumulation of LDL in the subendothelial space of arteries (44, 45). In these regions, LDL becomes susceptible to oxidation by ROS, forming ox-LDL (46). ox-LDL not only exhibits pro-inflammatory properties but also activates various immune responses (46). ox-LDL binds to scavenger receptors on endothelial cell (EC), macrophages, and smooth muscle cells, triggering intracellular signaling pathways that initiate inflammatory responses (47). For instance, ox-LDL activates the Nuclear Factor kappa-light-chain-enhancer of activated B cells (NF-*κ*B) pathway, promoting the expression of various pro-inflammatory cytokines and chemokines like IL-6 and TNF-α (47, 48). Ox-LDL has different effects on vascular smooth muscle cells (VSMCs) and ECs (49, 50). In ECs, ox-LDL activates the NF-κB pathway through Toll-like receptors (TLRs) and other receptors, increasing the expression of pro-inflammatory cytokines such as IL-6, Interleukin-8 (IL-8), and Monocyte Chemoattractant Protein-1 (MCP-1), which attract monocytes to the vessel wall and promote plaque formation (51). In VSMCs, ox-LDL also activates the NF-κB pathway but primarily promotes cell proliferation and migration, influencing plaque stability or instability (52). Additionally, the phenotypic switch of VSMCs from a contractile to a synthetic phenotype is associated with NF-κB activation (52).

### Inflammation amplification and immune cell recruitment

The presence of ox-LDL and lipid accumulation in the arterial wall further induces the overproduction of pro-inflammatory cytokines (such as IL-6 and TNF-α) and chemokines, such as Chemokine (C-C motif) ligand 2 (CCL2) and Chemokine (C-X-C motif) ligand 10 (CXCL10) (53, 54). These molecules bind to their respective receptors on EC and immune cells, further amplifying the inflammatory response. IL-6 and TNF-α activate the Janus Kinase—Signal Transducer and Activator of Transcription (JAK-STAT) and NF-*κ*B signaling pathways, promoting inflammatory responses and stimulating the proliferation and migration of vascular wall cells (55, 56). Chemokines (like CCL2) recruit monocytes to the arterial wall, enhancing the local immune response (57). CCL2 binds to the Chemokine (C-C motif) receptor 2 (CCR2) on monocytes, facilitating their adhesion to the endothelium and subsequent infiltration into the vascular wall (57). As atherosclerosis progresses, the accumulation of cholesterol and its metabolites triggers additional inflammatory responses by activating the NLR family, pyrin domain containing 3 (NLRP3) inflammasome, a key intracellular inflammatory signaling complex (58). Activation of the NLRP3 inflammasome leads to Cysteine-aspartic protease 1 (caspase-1) activation, which promotes the conversion of Pro-Interleukin-1β (pro-IL-1β) and Pro-Interleukin-18 (pro-IL-18) into their mature forms, Interleukin-1β (IL-1β) and Interleukin-18 (IL-18) (59, 60). These cytokines further amplify local inflammation and induce apoptosis and necrosis of vascular wall cells (60). Activation of the inflammasome is also associated with plaque instability, potentially leading to plaque rupture and thrombosis, which can trigger acute cardiovascular events such as myocardial infarction and stroke (61).

### Endothelial cell activation and foam cell formation

In the early stages of atherosclerosis, EC become activated due to various risk factors such as ox-LDL, hyperglycemia, and hypertension (62). Activated EC express various adhesion molecules, including intercellular adhesion molecule-1 (ICAM-1), vascular cell adhesion molecule-1 (VCAM-1), and Endothelial Selectin (E-selectin) (63). The upregulation of ICAM-1 and VCAM-1 is a hallmark of endothelial cell activation (64). These molecules bind to integrins on monocytes, promoting firm adhesion of monocytes to the endothelium and their subsequent infiltration into the subendothelial space (65, 66). Endothelial cell activation also involves the release of pro-inflammatory cytokines like IL-1β and TNF-α, which further enhance endothelial adhesion and permeability, promoting monocyte migration (67). Monocytes that adhere to activated EC, guided by chemokines, cross the endothelial layer and infiltrate the intima (68). Once in the intima, monocytes differentiate into macrophages, which then phagocytose large amounts of ox-LDL through scavenger receptors (47). These macrophages, laden with excessive lipid, transform into foam cells (69). The formation of foam cells is a hallmark of atherosclerotic plaques and involves the release of various inflammatory mediators, further promoting the development of local inflammation (69, 70). The apoptosis and death of foam cells contribute to plaque instability by releasing large amounts of lipids and cellular debris, increasing the risk of plaque rupture and thrombosis (71).

### Smooth muscle cell phenotypic switching and changes in plaque stability

**Figure 1:**
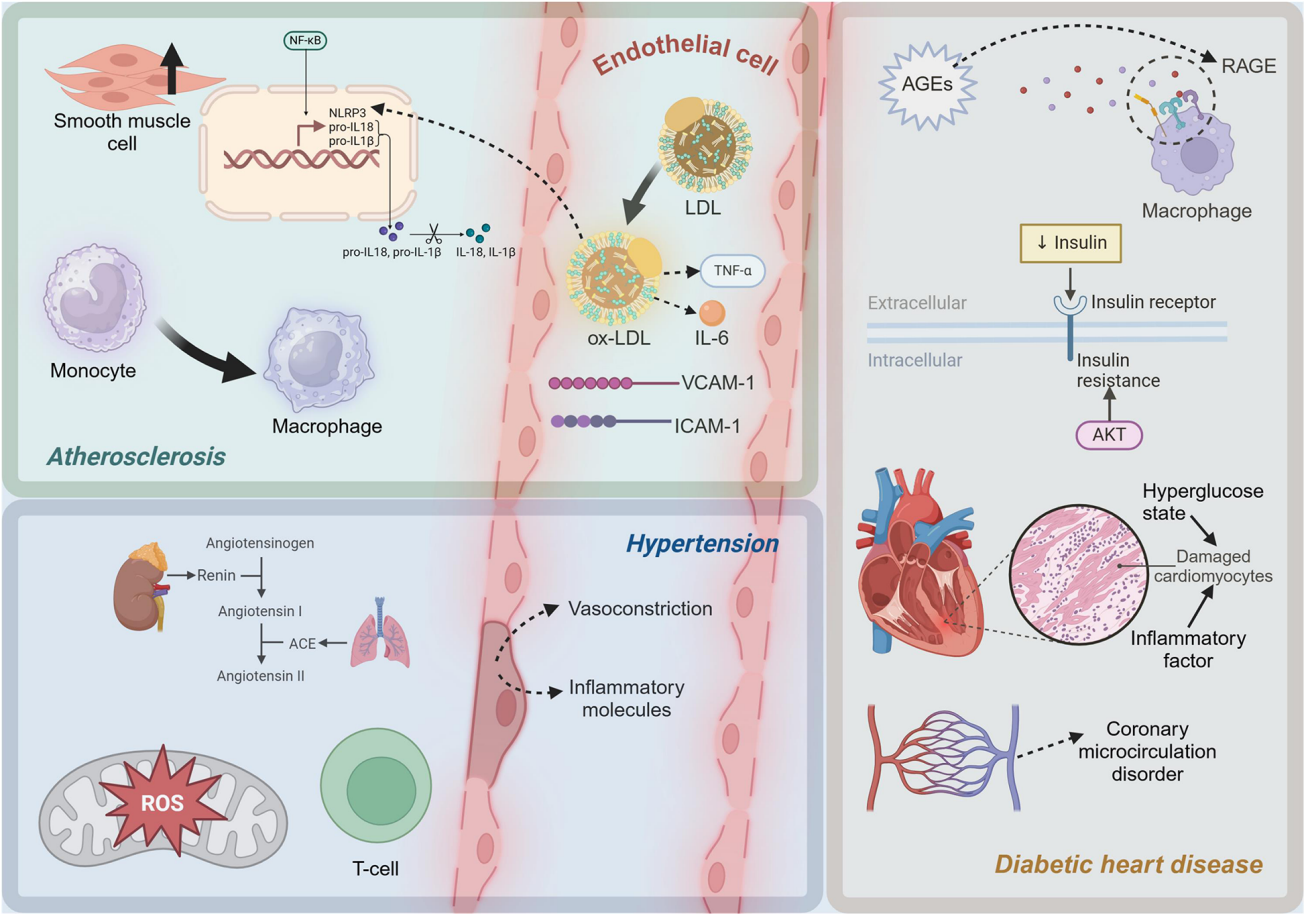
Diagram of inflammatory mechanisms in cardiometabolic diseases. LDL, low-density lipoprotein; ox-LDL, oxidized low-density lipoprotein; TNF-α, Tumor Necrosis Factor-alpha; IL-6, Interleukin-6; ICAM-1, intercellular adhesion molecule-1; VCAM-1, vascular cell adhesion molecule-1; NF-*κ*B, Nuclear Factor kappa-light-chain-enhancer of activated B cells; NLRP3, NLR family, pyrin domain containing 3; pro-IL-1β, Pro-Interleukin-1β; pro-IL-18, Pro-Interleukin-18; IL-1β, Interleukin-1β; IL-18, Interleukin-18; ROS, reactive oxygen species; ACE, Angiotensin-Converting Enzyme; AGEs, advanced glycation end products; RAGE, Receptor for Advanced Glycation Endproducts. This figure was created with BioRender.com.

In the progression of atherosclerosis, smooth muscle cells undergo proliferation and a shift towards a synthetic phenotype under the influence of inflammatory mediators (72). This process involves a phenotypic change that enables smooth muscle cells to produce extracellular matrix components such as collagen, forming fibrous caps and thickening the arterial intima (73). Smooth muscle cell proliferation and synthetic phenotype transformation play a critical role in stabilizing plaques (74). However, as the disease progresses, the apoptosis and necrosis of smooth muscle cells can lead to the rupture of fibrous caps, increasing plaque instability and the risk of cardiovascular events (75). In summary, the inflammatory mechanisms of atherosclerosis involve complex interactions among multiple cells and molecules. In Figure 1, the molecules and cells involved in the inflammatory microenvironment of atherosclerosis can be seen. The oxidation of LDL, the release of pro-inflammatory cytokines, the role of cholesterol metabolites, and the dynamic changes in EC, monocytes, and smooth muscle cells all contribute significantly to the progression of this disease.

## Inflammatory mechanisms in hypertension

### Role of RAS in hypertension-related inflammation

Hypertension is a complex chronic disease with pathophysiological mechanisms involving multiple aspects, with inflammatory mechanisms receiving increasing attention in recent years (76, 77). Inflammation not only serves as a consequence of hypertension but also plays a crucial role in its progression and maintenance. The Renin-Angiotensin System (RAS) plays a pivotal role in the pathogenesis and progression of hypertension, with Angiotensin II (Ang II) being the key effector molecule (78). Ang II, by binding to the Angiotensin II type 1 receptor (AT1R), activates multiple signaling pathways, leading to vasoconstriction, sodium and water retention, and increased blood pressure (79). These inflammatory mediators further attract and activate immune cells, leading to vascular wall inflammation. ROS, including superoxide, hydrogen peroxide, and hydroxyl radicals, play significant roles in the inflammatory mechanisms of hypertension (80). Ang II increases ROS production via the nicotinamide adenine dinucleotide phosphate (NADPH) oxidase system, which can directly damage vascular EC and activate inflammatory signaling pathways (81). In hypertensive patients, the activity of antioxidant enzymes such as superoxide dismutase (SOD) and glutathione peroxidase (GPx) often decreases, leading to weakened ROS clearance and further exacerbating oxidative stress and inflammation (82).

### Oxidative stress and endothelial damage

Endothelial dysfunction is a critical aspect of inflammatory mechanisms in hypertension (83). Upon exposure to Ang II, ROS, and other stimuli, EC release molecules like endothelin-1 (ET-1) and VCAM-1, which promote vasoconstriction and increase leukocyte adhesion and infiltration, leading to vascular wall inflammation (84, 85). Under normal conditions, EC produce nitric oxide (NO) through endothelial nitric oxide synthase (eNOS), which has vasodilatory and anti-inflammatory effects (86, 87). However, in hypertension, eNOS activity declines, reducing NO production, resulting in vasoconstriction and heightened inflammation (88). The immune system plays a crucial role in the inflammatory mechanisms of hypertension, with lymphocytes, particularly T cells, being significantly involved (89). In hypertensive patients and animal models, there is significant infiltration of T cells around blood vessels and in renal tissues (90). These T cells release cytokines such as interferon-*γ* (IFN-*γ*) and interleukin-17 (IL-17), further promoting local inflammatory responses (91). T Helper 1 (Th1) cells primarily secrete IFN-γ, which activates macrophages and promotes inflammation; T Helper 17 (Th17) cells mainly secrete IL-17, which is involved in neutrophil recruitment and activation (91, 92). These cytokines work together to cause chronic inflammation in blood vessels and kidneys, further maintaining and exacerbating hypertension.

The inflammatory mechanisms in hypertension are a complex process involving multiple interactions at both molecular and cellular levels. Figure 1 shows the molecules and cells involved in the inflammatory microenvironment of hypertension. At the molecular level, activation of the RAS, particularly the pro-inflammatory effects of Ang II, and exacerbation of oxidative stress, are crucial drivers of inflammation. At the cellular level, endothelial dysfunction and the involvement of lymphocytes, particularly T cells, further promote local and systemic inflammatory responses. A deeper understanding of these mechanisms not only elucidates the pathophysiological basis of hypertension but also provides a theoretical foundation for developing new anti-inflammatory therapeutic strategies.

## Inflammatory mechanisms in diabetic heart disease

### Advanced glycation end products and inflammation in diabetic cardiomyopathy

Diabetes is classified into three primary types: type 1, type 2, and gestational diabetes (93–96). Diabetes is a long-term condition associated with various complications (97–105). Among these, diabetic cardiomyopathy (DCM) is a common and severe complication, characterized by myocardial dysfunction, cardiac remodeling, and heart failure (106). Inflammation plays a crucial role in the initiation and progression of DCM (107). In a hyperglycemic state, excess glucose can undergo non-enzymatic reactions with proteins, lipids, or nucleic acids to form advanced glycation end products (AGEs) (108). The formation of AGEs is a significant trigger of chronic inflammation in diabetic patients (109). In high glucose conditions, glucose reacts with proteins and lipids through the Maillard reaction to form Schiff bases, which then undergo Amadori rearrangement to produce stable AGEs (110). AGEs bind to their specific receptor, the receptor for advanced glycation end products (RAGE), and activate various pro-inflammatory signaling pathways (111). RAGE, a member of the immunoglobulin superfamily, is widely expressed in cardiomyocytes, EC, and macrophages (112). The binding of AGEs to RAGE can initiate multiple pro-inflammatory signaling cascades (113). Upon AGE-RAGE binding, the I*κ*B kinase (IKK) complex is activated, leading to the phosphorylation and degradation of the inhibitory protein Inhibitor of *κ*B alpha (I*κ*B*α*) (114). This releases NF-κB, enabling its translocation to the nucleus and initiating the transcription of pro-inflammatory genes such as TNF-α and IL-6 (114). The AGE-RAGE complex can also activate the mitogen-activated protein kinase (MAPK) pathway (115). These kinases promote the expression of inflammatory factors and contribute to apoptosis (115).

### Insulin resistance and oxidative stress in diabetic cardiomyopathy

Insulin resistance, a hallmark of T2D, is closely associated with inflammatory responses (116). Insulin resistance induces oxidative stress, leading to increased production of ROS, which in turn activates the expression of pro-inflammatory factors (117). In insulin-resistant states, the activity of NADPH oxidase and the mitochondrial electron transport chain increases, leading to elevated ROS production (118). ROS can activate multiple signaling pathways, including NF-*κ*B and MAPK, promoting the expression of pro-inflammatory cytokines such as TNF-α, IL-1β, and IL-6, which further exacerbate myocardial inflammation and damage (119). Myocardial cells are severely damaged under the dual impact of high glucose toxicity and inflammatory factors (120). A high glucose environment directly causes metabolic disturbances in cardiomyocytes, including imbalances in glycolysis and fatty acid oxidation, leading to energy metabolism disorders and cell damage (121). Pro-inflammatory cytokines such as TNF-α and IL-6 activate apoptotic signaling pathways, including the Caspase family and B-cell Lymphoma 2 (Bcl-2) family, leading to myocardial cell apoptosis (122). High glucose and AGEs can activate EC through the NF-κB pathway (123). The cardiac microvascular system in diabetic patients is severely affected by inflammation, leading to coronary microcirculatory disorders, which further exacerbate myocardial ischemia and injury (124). As depicted in Figure 1, in diabetic cardiomyopathy, a high-glucose environment induces the formation of AGEs, which activate pro-inflammatory pathways through the RAGE. Additionally, insulin resistance increases pro-inflammatory factors related to oxidative stress. Myocardial cells are damaged, and microvascular inflammation leads to coronary microcirculatory dysfunction, exacerbating heart injury.

### The role of gut microbiota in obesity-related cardiovascular metabolic disorders

Obesity-related CMD are closely associated with chronic low-grade inflammation (125). Key mechanisms include inflammation in adipose tissue, insulin resistance, and cardiac inflammation (126). Recent research has highlighted the role of gut microbiota in the inflammatory microenvironment of CMD (127). Impaired gut barrier function, reduced production of short-chain fatty acids (SCFAs), and increased generation of microbial metabolites like trimethylamine N-oxide (TMAO) are all linked to the development and progression of CMD (128, 129). Future research needs to further explore individual variations in gut microbiota, interactions with the host immune system, and potential interventions based on gut microbiota.

### Systems biology integration of multi-omics data to elucidate mechanisms of CMD inflammatory microenvironment

From a systems biology perspective, integrating multi-omics data can provide a comprehensive theoretical framework and data support for elucidating the mechanisms underlying the formation of the inflammatory microenvironment in CMD. Firstly, genomics analysis can reveal genetic variations associated with CMD inflammation and identify potential risk genes (130). Secondly, proteomics data can elucidate the functions and interaction networks of key proteins in the inflammatory microenvironment, revealing the roles of proteins in signal transduction and functional regulation (131). Simultaneously, metabolomics research can capture the dynamic changes in metabolites during the inflammatory process, revealing the critical roles of metabolic pathways in inflammation regulation (132). This integrated analysis not only helps to uncover the complex regulatory network of the CMD inflammatory microenvironment but also provides a theoretical foundation and experimental basis for developing precision intervention strategies based on specific targets, thereby promoting the advancement of CMD-related research to a deeper level.

## Conclusion

This review has explored the complex inflammatory mechanisms in CMD diseases, including atherosclerosis, hypertension, and DCM. These conditions involve intricate interactions among various inflammatory factors, cell types, and signaling pathways. In atherosclerosis, ox-LDL and cytokines like IL-6 and TNF-α drive foam cell formation and arterial thickening. Hypertension involves RAS activation, oxidative stress, and T cell-mediated inflammation. DCM results from AGEs accumulation, RAGE activation, and cardiac inflammation induced by high glucose levels. By understanding these mechanisms, we can develop targeted treatments that manage and potentially reverse disease progression. Personalized strategies based on specific inflammatory pathways are crucial for improving patient outcomes, and precision medicine offers promising avenues to enhance the quality of life and prognosis for individuals with these conditions.

## Glossary

CMD: Cardiometabolic diseases
ox-LDL: oxidized low-density lipoprotein
IL-6: Interleukin-6
TNF-α: Tumor Necrosis Factor-alpha
RAS: renin-angiotensin system
AGEs: advanced glycation end products
RAGE: Receptor for Advanced Glycation Endproducts
MetS: metabolic syndrome
CVD: Cardiovascular disease
MR: Mendelian randomization
T2D: type 2 diabetes
SIRT1: Silent Information Regulator 2 Ortholog 1
sdLDL-C: small dense LDL cholesterol
RLP-C: remnant-like particle cholesterol
VSMCs: vascular smooth muscle cells
EC: endothelial cells
TLRs: Toll-like receptors
IL-8: Interleukin-8
MCP-1: Monocyte Chemoattractant Protein-1
LDL-C: low-density lipoprotein cholesterol
ROS: reactive oxygen species
DNA: Deoxyribonucleic Acid
LDL: low-density lipoprotein
FFA: free fatty acids
NF-κB: Nuclear Factor kappa-light-chain-enhancer of activated B cells
CCL2: Chemokine (C-C motif) ligand 2
CXCL10: Chemokine (C-X-C motif) ligand 10
JAK-STAT: Janus Kinase—Signal Transducer and Activator of Transcription
CCR2: Chemokine (C-C motif) receptor 2
NLRP3: NLR family, pyrin domain containing 3
caspase-1: Cysteine-aspartic protease 1
pro-IL-1β: Pro-Interleukin-1β
pro-IL-18: Pro-Interleukin-18
IL-1β: Interleukin-1β
IL-18: Interleukin-18
ICAM-1: intercellular adhesion molecule-1
VCAM-1: vascular cell adhesion molecule-1
E-selectin: Endothelial Selectin
Ang II: Angiotensin II
AT1R: Angiotensin II type 1 receptor
NADPH: nicotinamide adenine dinucleotide phosphate
SOD: superoxide dismutase
GPx: glutathione peroxidase
ET-1: endothelin-1
NO: nitric oxide
eNOS: nitric oxide synthase
IFN-*γ*: interferon-*γ*
IL-17: interleukin-17
Th1: T Helper 1
Th17: T Helper 17
DCM: Diabetic cardiomyopathy
IKK: I*κ*B kinase
I*κ*B*α*: inhibitory protein Inhibitor of *κ*B alpha
MAPK: mitogen-activated protein kinase
Bcl-2: B-cell Lymphoma 2
SCFAs: short-chain fatty acids
TMAO: trimethylamine N-oxide.
